# Exogenous H_2_S Promoted USP8 Sulfhydration to Regulate Mitophagy in the Hearts of db/db Mice

**DOI:** 10.14336/AD.2019.0524

**Published:** 2020-03-09

**Authors:** Yu Sun, Fanghao Lu, Xiangjing Yu, Bingzhu Wang, Jian Chen, Fangping Lu, Shuo Peng, Xiaojiao Sun, Miao Yu, He Chen, Yan Wang, Linxue Zhang, Ning Liu, Haining Du, Dechao Zhao, Weihua Zhang

**Affiliations:** ^1^Department of Pathophysiology, Harbin Medical University, Harbin, China.; ^2^Department of Forensic Medicine, Harbin Medical University, Harbin, China.; ^3^Department of Urologic Surgery, First affiliated hospital of Harbin Medical University, Harbin, China.; ^4^Department of Cardiology, First affiliated hospital of Harbin Medical University, Harbin, China.; ^5^Key Laboratory of Cardiovascular Medicine Research (Harbin Medical University), Ministry of Education, Harbin, China

**Keywords:** diabetic cardiomyopathy, hydrogen sulfide, mitophagy, USP8, Parkin

## Abstract

Hydrogen sulfide (H_2_S), an important gasotransmitter, regulates cardiovascular functions. Mitochondrial damage induced by the overproduction of reactive oxygen species (ROS) results in myocardial injury with a diabetic state. The purpose of this study was to investigate the effects of exogenous H_2_S on mitophagy formation in diabetic cardiomyopathy. In this study, we found that exogenous H_2_S could improve cardiac functions, reduce mitochondrial fragments and ROS levels, enhance mitochondrial respiration chain activities and inhibit mitochondrial apoptosis in the hearts of db/db mice. Our results showed that exogenous H_2_S facilitated parkin translocation into mitochondria and promoted mitophagy formation in the hearts of db/db mice. Our studies further revealed that the ubiquitination level of cytosolic parkin was increased and the expression of USP8, a deubiquitinating enzyme, was decreased in db/db cardiac tissues. S-sulfhydration is a novel posttranslational modification of specific cysteine residues on target proteins by H_2_S. Our results showed that the S-sulfhydration level of USP8 was obviously decreased in vivo and in vitro under hyperglycemia and hyperlipidemia, however, exogenous H_2_S could reverse this effect and promote USP8/parkin interaction. Dithiothreitol, a reducing agent that reverses sulfhydration-mediated covalent modification, increased the ubiquitylation level of parkin, abolished the effects of exogenous H_2_S on USP8 deubiquitylation and suppressed the interaction of USP8 with parkin in neonatal rat cardiomyocytes treated with high glucose, oleate and palmitate. Our findings suggested that H_2_S promoted mitophagy formation by increasing S-sulfhydration of USP8, which enhanced deubiquitination of parkin through the recruitment of parkin in mitochondria.

Large epidemiological studies have confirmed that type 2 diabetes is associated with increased mortality caused by augmented risk of cardiovascular death [1]. In diabetes, mitochondria are a predominant source of intracellular reactive oxygen species (ROS) and are the primary target of oxidative injury [2]. Damaged mitochondria can further increase ROS production through ROS-induced ROS release and may induce leakage of pro-death factors, induce cardiomyocyte death. In physiologic state, cells can remove damaged mitochondria to prevent the accumulation of ROS and this process of mitochondrial quality control is mediated by mitophagy, the selective autophagic removal of damaged mitochondria[3]. Mitochondrial dynamics have been reported to play an important role in mitophagy in various diseases[4]. The mitochondrial fusion and fission cycle are proposed to balance two competing processes: compensation of damage by fusion and elimination of damage by fission. The gradual accumulation of damaged components poses a problem for the mitophagic disposal process [5].

Evidence is emerging that a specific group of mitochondrial proteins that have been linked to familial forms of Parkinson's disease (PD), may provide novel therapeutic targets for cardioprotection [6]. Parkin is a PD-associated protein that occupies a pivotal position in cellular biology because a loss or gain of its function drives abnormal cellular responses that lead to cell death in neurodegenerative disease [7]. Parkin is an RBR-type E3 ubiquitin-ligase that localizes in the cytosol as an autoinhibited form and its activity is critical for the efficient elimination of dysfunctional mitochondria by mitophagy [8]. Ubiquitin (Ub) plays important roles in many different cellular functions, including protein degradation, signaling, endocytosis, and the immune system, and it also serves as an important regulator of mitochondrial dynamics [9, 10]. Auto-ubiquitination of parkin can be antagonized by deubiquitinating enzymes (DUBs), which remove Ub from the E3 [11]. Previous studies have demonstrated that USP8 (Ubiquitin Specific Peptidase 8) is a DUB that preferentially removes non-canonical K6-linked ubiquitin chains from parkin, a process required for the efficient recruitment of parkin to depolarized mitochondria [12]. Severe damage or the depolarization of mitochondria induces the recruitment of parkin to the mitochondrial surface, where it ubiquitylates proteins in the mitochondrial outer membrane, initiating their proteasomal degradation and culminating in mitophagy, the selective autophagic removal of the whole organelle.

Hydrogen sulfide (H_2_S), as the third gasotransmitter, plays diverse physiological and pathological roles in the body [13]. It is widely recognized that H_2_S can directly affect blood pressure, promote vasorelaxation, inhibit monocyte adhesion and induce angiogenesis[14]. H_2_S levels in serum were reduced and may be involved in the progression of diabetes [15]. Our present study investigated whether exogenous H_2_S could protect myocardiocytes by promoting mitophagy in type 2 diabetes. The effect of exogenous H_2_S might contribute to upregulating USP8 expression, promoting the recruitment of parkin in mitochondria and facilitating the parkin-mediated clearance of Ub aggregates via mitophagy. Therefore, we speculated that exogenous H_2_S likely promoted the USP8-mediated deubiquitination of parkin that regulated mitochondrial function via promoting mitophagy under hyperglycemia and hyperlipidemia.

## MATERIALS AND METHODS

### Diabetes model and treatment protocols

Homozygous male and female ten-week-old db/db mice on a C57BL/6 background (n=50) and their corresponding wild-type (n=30) littermates were used in this study. All mice were provided by the Animal Laboratory Center of Nanjing University. Animals were housed in a climate- and temperature-controlled room on a 12:12 h light-dark cycle. The mice were maintained on a standard diet and water ad libitum. Half of the db/db mice were placed in the NaHS treatment group and treated with NaHS (80 μmol/kg) by intraperitoneal injection every 2 days for twelve weeks. All animal experiments were performed according to the Guide for the Care and Use of Laboratory Animals published by the China National Institutes of Health and were approved by the Animal Care Committees of Harbin Medical University, China.

### Morphological changes in the cardiac tissues of experimental mice

Ultrastructural alterations in cardiac tissues were detected by transmission electron microscopy (TEM). Cardiac tissues for TEM were cut into pieces less than 1 mm^3^ and fixed in 2.5% glutaraldehyde in 0.1 M sodium cacodylate buffer (pH 7.4) for 4 h. Tissues were postfixed in osmium tetroxide and embedded in Epon 812(Electron Microscopy Sciences). Ultrathin sections were stained with uranyl acetate and lead citrate and examined under a Zeiss Axiophot microscope.

### Neonatal rat cardiomyocytes culture

Primary cultures of neonatal rat cardiomyocytes were prepared by previously described methods [16]. Neonatal rat cardiomyocytes were prepared from two- to three-day-old neonatal Wistar rats (Animal Research Institute of Harbin Medical University, China). Then, the hearts were cut into pieces of less than 1 mm^3^ and incubated with 0.25% trypsin for 8 min at 37°C for six times. The supernatant cells were collected and then isolated by centrifugation for 10 min at 2000 g at room temperature. The cells were resuspended in DMEM containing 10% (v/v) fetal bovine serum (HyClone), 100 U/mL penicillin and 100 mg/mL streptomycin and were cultured in a humidified atmosphere containing 5% CO_2_ at 37°C. After 1 h hour of incubation at 37°C, the attached cells were discarded, and the unattached cells were cultured in new media. The media were replaced every two days.

### Cellular experimental protocol

The cultured neonatal rat cardiomyocytes were randomly divided into the following groups and treatments: control group (low glucose, LG, 5.5 mM), high glucose (HG, 40 mM) +Oleate (Ole, 200 µM)+ Palmitate (Pal, 200 µM), HG+Ole+Pal+NaHS (100 µM, H_2_S doner), HG+Ole+ Pal+Mito-tempo (2 µM, an inhibitor of mitochondrial ROS), HG+Ole+Pal+Mdivi-1 (50 µM, an inhibitor of Drp1), HG+Ole+Pal+ Bafilomycin A1 (100 nM, an inhibitor of autophagy), HG+Ole+Pal+NaHS+ Bafilomycin A1, HG+Ole+Pal+DTT (1 mM, an inhibitor of disulfide bond), HG+Ole+Pal+DTT+NaHS, HG+Ole +Pal+PPG (10 nM, an irreversible competitive CSE inhibitor). Drugs were added directly in cultured medium for 48 h. Neonatal rat cardiomyocytes treated with high glucose and palmitate and oleate classically mimic hyperglycemia and hyperlipidemia. NaHS, palmitate, oleate, PPG, DTT, Mito-TEMPO and Mdivi-1 were purchased from Sigma-Aldrich (Sigma). Bafilomycin A1 was purchased from MedChem Express (MCE).

### Fat-overloading induction in neonatal rat cardiomyocytes

To induce fat-overloading in cells, primary cultures of neonatal rat cardiomyocytes at 75% confluency were exposed to a mixture of long-chain of free fatty acids (FFAs: oleate and palmitate). Stock solutions of 5 mM oleate acid (Sigma, USA) and 5 mM palmitate (Sigma, USA) prepared in culture medium containing 1% bovine serum albumin (BSA) were conveniently diluted in culture medium to obtain the desired final concentrations. The FFA mixture was added to neonatal rat cardiomyocytes for 48 h.

### Palmitate/BSA solution preparation

The palmitate solution used for incubation with neonatal rat cardiomyocytes as previously described with slight modifications [17, 18]. A 100 mM palmitate stock solution was prepared in 100 mM NaOH by heating at 70 °C. At 55 °C, a 10% (wt/v) FFAs-BSA solution was prepared in PBS. A total of 5 mL of the 100 mM palmitate solution was added dropwise to 95 mL 10% BSA solution at 55 °C in a shaking water bath. Then, the solution was mixe with a vortex for 10 s followed by 10-min incubation. A stock solution of 5 mM palmitate was cooled to room temperature and sterile filtered (0.22 μm pore size membrane filter).

### Oleate/BSA solution preparation

A 5 mM oleate stock solution was prepared in 10% BSA solution at room temperature and sterile filtered (0.22 μm pore size membrane filter).

### Mitochondria isolation

The cardiac tissues (n=6, per group) and neonatal rat cardiomyocytes were washed twice with ice-cold PBS resuspended in lysis buffer (mM: 20 HEPES/KOH, pH 7.5, 10 KCl, 1.5 MgCl_2_, 1.0 sodium EDTA, 1.0 sodium EGTA, 1.0 DTT, 0.1 PMSF, and 250 sucrose), and then homogenized with a homogenizer in ice/water. After removing the nuclei and cell debris by centrifugation at 1000 g for 10 min at 4°C, the supernatants were further centrifuged at 10000 g for 10 min at 4°C. The resulting mitochondrial pellets were resuspended in lysis buffer. The supernatants and mitochondrial fractions were stored at -80°C.

### Immunoblot analysis

Western blotting was performed as described previously. The primary antibodies included anti-USP8 (Proteintech, USA), anti-PINK1 (Proteintech, USA), anti-Parkin(CST, USA), anti-Ubiquitin (Proteintech, USA), anti-GAPDH (Proteintech, USA), anti-VDAC1 (Proteintech, USA), anti-Fis1 (Proteintech, USA), anti-Drp1 (CST, USA), anti-phospho-Drp1 (Ser616, CST, USA), anti-Bax, anti-Bcl2, anti-Mfn2, anti-SOD, anti-Mn-SOD, anti-ATG7, anti-Beclin1 (all were from Proteintech, USA), anti-P62 (CST, USA), anti-LC3II/I (Proteintech, USA), anti-LC3B (Proteintech, USA), anti-CAT (Proteintech, USA), anti- cytochrome C (Proteintech, USA), anti-Cleaved-caspase9 (Proteintech, USA). Densitometry was conducted with the image processing and analysis program AlphaView.SA, and the data were expressed as relative units.

### Immunoprecipitation

The cells were harvested and lysed as previously described. Antibodies specific to USP8 or PINK1 were added to the supernatants, and the mixture was incubated. Each sample was then precipitated with protein A agarose beads. Bound proteins were eluted by boiling with loading buffer and analyzed by Western blotting with anti-Parkin antibody.

### Analysis of mitochondrial transmembrane potential

Changes in mitochondrial transmembrane potential were assessed using the lipophilic cationic probe 5,5',6,6'-tetrachloro-1,1',3,3'-tetraethyl-imida-carbocyanine iodide (JC-1). Neonatal rat cardiomyocytes were seeded and treated for 48 h at 37°C. After experimentation, cells were loaded with 2 µM JC-1 (Invitrogen, USA) at 37°C in the dark for 30 min and washed three times with cold PBS. Green fluorescence indicated the monomeric form of JC-1, and red fluorescence indicated the aggregated form. The cells were monitored using a fluorescence microscope (Olympus, XSZ-D2).

### Mitochondrial fragmentation

Using MitoTracker staining (Beyotime, China) to observe the mitochondrial morphology, neonatal rat cardio-myocytes were seeded in 35 mm culture dishes and treated with different reagents and 200 nM MitoTracker for 30 min in a 37 °C incubator containing 5% CO_2_. Then, the cells were washed with PBS. Fluorescence microscopy (Olympus, XSZ-D2) was used for visualization and to determine the fluorescence intensity. We subtracted the background fluorescence from the acquired images, the images were then filtered, and binary operations were applied to identify mitochondrial segments using ImageJ (NIH Bethesda, MD). The continuous mitochondrial structures were counted, and the number was normalized to the total mitochondrial area to obtain the mitochondrial fragmentation count (MFC) for each group of 25 or more randomly selected cells, as described previously. Cells with greater fragmentation exhibit a higher MFC. The mitochondrial lengths were measured using NIS Elements software and scored as follows: fragmented (globular, <2 µm diameter); intermediate (2-4 µm long); and filamentous (>4 µm long). Approximately 200 cells were analyzed, and the experiments were performed in triplicate by two individuals.

### Mitochondrial autophagosome detection

Neonatal rat cardiomyocytes were cultured in 24-well plates. Mitochondrial autophagosomes were detected according to the assay protocol (Mitophagy detection kit, Dojindo, Japan). Mitophagy dye accumulates in intact mitochondria, is immobilized on the intact mitochondria with chemical bonds and exhibits weak fluorescence from the influence of surrounding conditions. When mitophagy is induced, the damaged mitochondria fuse to lysosome, and then the mitophagy dye emits marked fluorescent signals. After the cells were incubated with a 100 nmol/l mitophagy dye working solution at 37°C for 30 min, cells were treated with HG+ Ole+Pal, HG+Ole+Pal+NaHS, HG+ Ole+Pal+Mito- tempo, HG+ Ole+Pal+Bafilomycin A1, and HG+ Ole+ Pal+NaHS+Bafilomycin A1 for 48 h. Then, cells were incubated at 37°C for 30 min with 1 µM Lyso dye working solution to observe the colocalization of the mitophagy dye and lysosomes. The mitophagy phenomenon and the fusion of mitochondria with lysosomes were observed by fluorescence microscope (Olympus, XSZ-D2).

### MDC assay for visualization of autophagic vacuoles

Monodansylcadaverine (MDC, Solarbio, China) has autofluorescence properties with an excitation wavelength at 365 nm, due to a dansyl group conjugated to cadaverine, a diamine-pentane. Under in vivo conditions, MDC accumulates as a selective fluorescent marker for autophagic vacuoles by interacting with membrane lipids that are highly concentrated in autophagic compartments. When MDC is incorporated into cells, the accumulation of this fluorescent reagent is observed in spherical compartments at the perinuclear region in spots distributed throughout the cytoplasm. Neonatal rat cardiomyocytes were incubated with 50 μM MDC in PBS at 37°C for 30 min. Autophagic vacuoles were analyzed using fluorescence microscopy (Olympus, XSZ-D2).

### S-sulfhydration assay

The assay was carried out as described previously. Briefly, cells were homogenized in HEN buffer [250 mM HEPES-NaOH (pH 7.7), 1 mM EDTA, and 0.1 mM neocuproine] supplemented with 100 μM deferoxamine and centrifuged at 13,000 g for 30 min at 4°C. Cell lysates (240 μg) were added to the blocking buffer [HEN buffer adjusted to 2.5% SDS and 20 mM methyl methanethiosulfonate (MMTS)] at 50°C for 20 min with frequent vortexing. The MMTS was then removed by acetone and the proteins were precipitated at -20°C for 20 min. After acetone removal, the proteins were resuspended in HENS buffer (HEN buffer adjusted to 1% SDS). To the suspension was added to 4 mM biotin-HPDP in dimethyl sulfoxide without ascorbic acid. After incubation for 3 h at 25°C, biotinylated proteins were precipitated by streptavidin-agarose beads, which were then washed with HENS buffer. The biotinylated proteins were eluted by SDS-polyacrylamide gel electrophoresis (SDS-PAGE) sample buffer and subjected to Western blot analysis. EDTA, neocuproine, deferoxamine, SDS, MMTS, biotin-HPDP, and streptavidin-agarose beads were purchased from Sigma-Aldrich (Sigma).

### Measurement of intracellular levels of polysulfide

The intracellular production of polysulfide was monitored using a newly developed fluorescent probe, SSP4 (Dojindo, Japan). Briefly, neonatal rat cardiomyocytes were loaded with 50 μM SSP4 in a serum-free DMEM containing 0.003% Cremophor EL for 15 min at 37°C in the dark. After being washing, SSP4 was measured using fluorescence microscope (Olympus, XSZ-D2).

### siRNA transfection

The neonatal rat cardiomyocytes (80% confluent) were treated according to the manufacturer’s instructions with USP8 short interfering RNAs (siRNAs) (mouse; Santa Cruz Biotechnology, USA) for 48 h to inhibit USP8 expression. The siRNA transfection of neonatal rat cardiomyocytes was achieved using Lipofectamine 2000(Invitrogen). Briefly, USP8-siRNA and the transfection reagent were incubated for 20 min to form complexes, which were then added to plates containing cells and medium. The cells were incubated at 37°C in a CO_2_ incubator for further analysis.

### Statistical analysis

The results were analyzed by using the Prism software package (GraphPad Software). The results are expressed as the mean ± standard deviation (SD). More than two groups were compared using a one-way ANOVA and Bonferroni’s correction. Differences between pairs of groups were analyzed using Student’s t-test.

**Figure 1.**
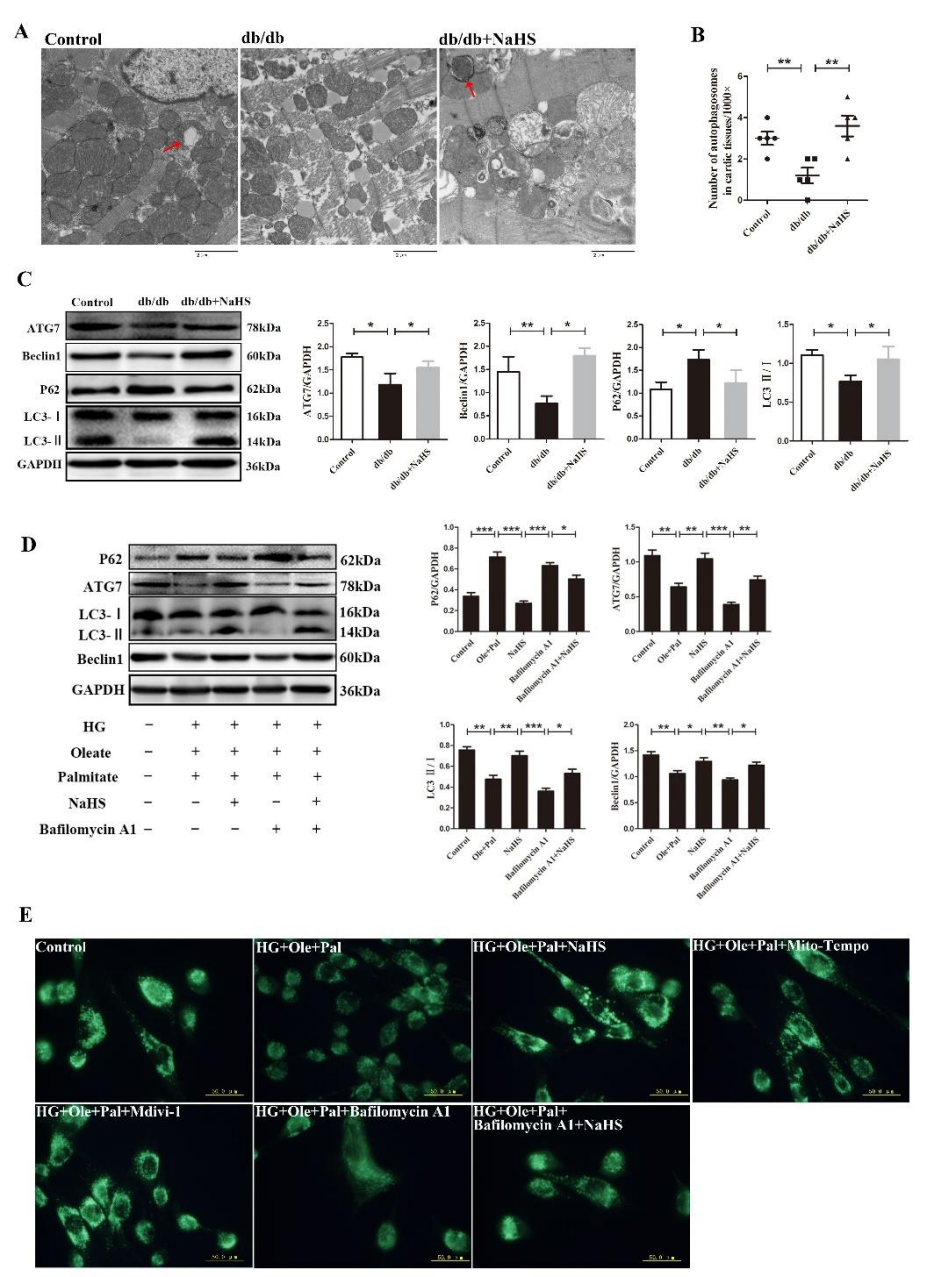
Exogenous H_2_S promoted autophagy in the hearts of db/db mice and in neonatal rat cardiomyocytes. (A) The ultrastructure of cardiac tissues was observed using a transmission electron microscope. The red arrow indicates mitophagosomes. (B) Data are presented as the number of autophagosomes in cardiac tissue in the control, db/db and db/db+NaHS groups (n=5). (C) The expression of Beclin1, Atg7 P62 and LC3II/I were examined in db/db cardiac tissues by western blotting. (D) The expression of Beclin1, ATG7, P62 and LC3II/I was examined by Western blotting following the treatment of Bafilomycin A1 in neonatal rat cardiomyocytes. (E) Autophagosomes were detected by the MDC test in neonatal rat cardiomyocytes (green). Values are presented as the mean ± S.D. from n = 5 replicates. *P<0.05, **P<0.01, ***P<0.001.

**Figure 2.**
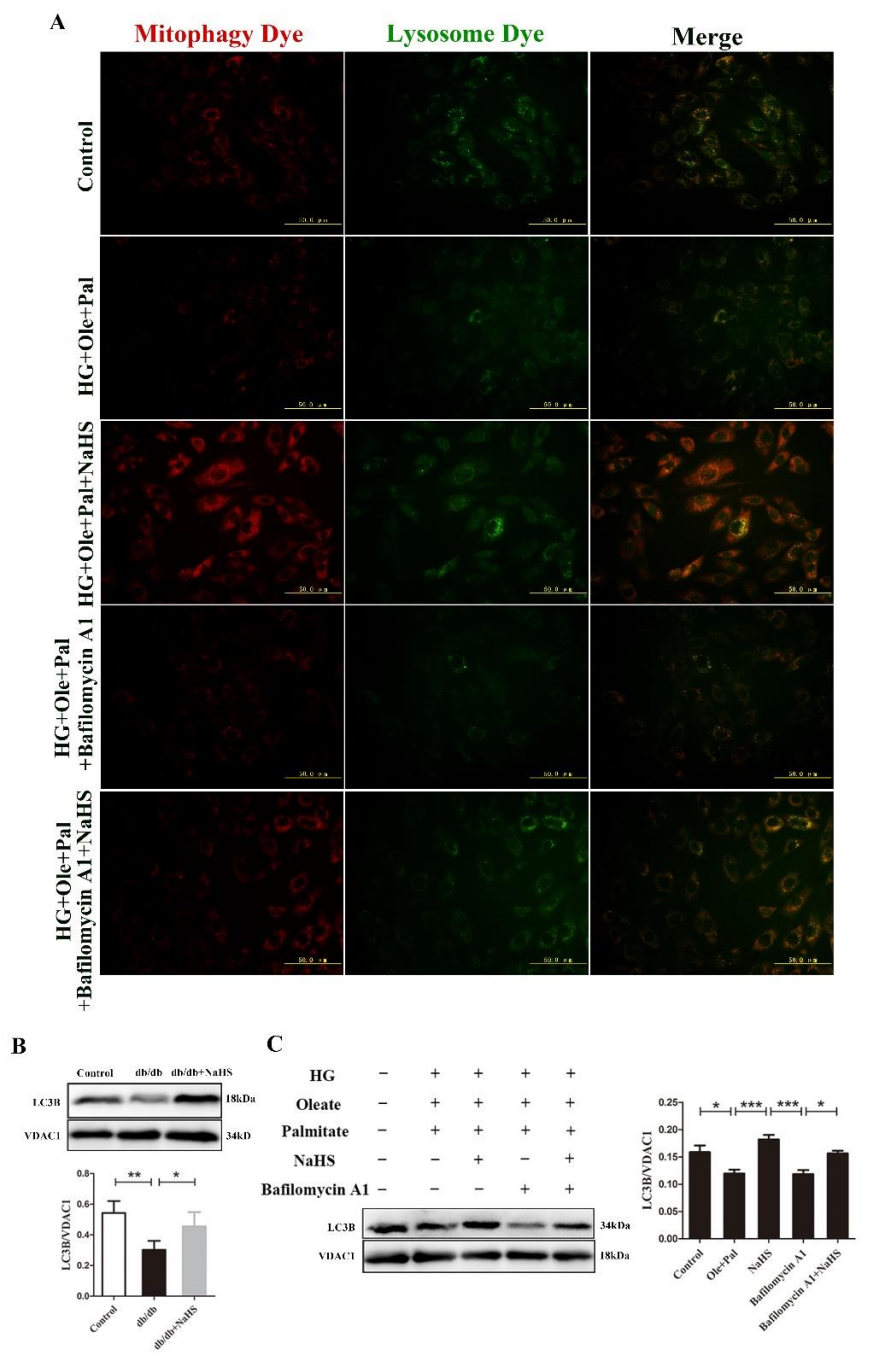
Exogenous H_2_S promoted mitophagy in the hearts of db/db mice and in neonatal rat cardiomyocytes. (A) Mitophagosomes were detected in neonatal rat cardiomyocytes by mitophagy detection kit. Red fluorescence represents the mitophagosomes and green fluorescence represents the fusion of mitophagosomes and lysosomes. (B) The expression of LC3B was examined in the mitochondria of db/db cardiac by Western blotting. (C) The expression of LC3B in mitochondria was examined by western blotting following the treatment of neonatal rat cardiomyocytes with Bafilomycin A1. Values are presented as the mean ± S.D. from n = 4 replicates. *P<0.05, **P<0.01, ***P<0.001.

## RESULTS

### Exogenous H_2_S improved cardiac function in db/db mice

In our study, db/db mice, leptin receptor-deficient mice, were chosen as the type 2 diabetes animal model. The corresponding wild-type littermates were used as the control group. The db/db mice and wild type mice were intraperitoneallly injected with 80 μmol/kg NaHS every 2 days for twelve weeks respectively. Our data showed that the body weight and plasma glucose levels of the db/db mice were significantly higher than those of the control mice at different time points. We also found that glucose intolerance, plasma insulin and plasma triglyceride levels in 22-week-old db/db mice were increased in db/db mice compared to db/db mice treated with NaHS, recapitulating the hallmark features of type 2 diabetes (Supplementary Fig. 1).

**Figure 3.**
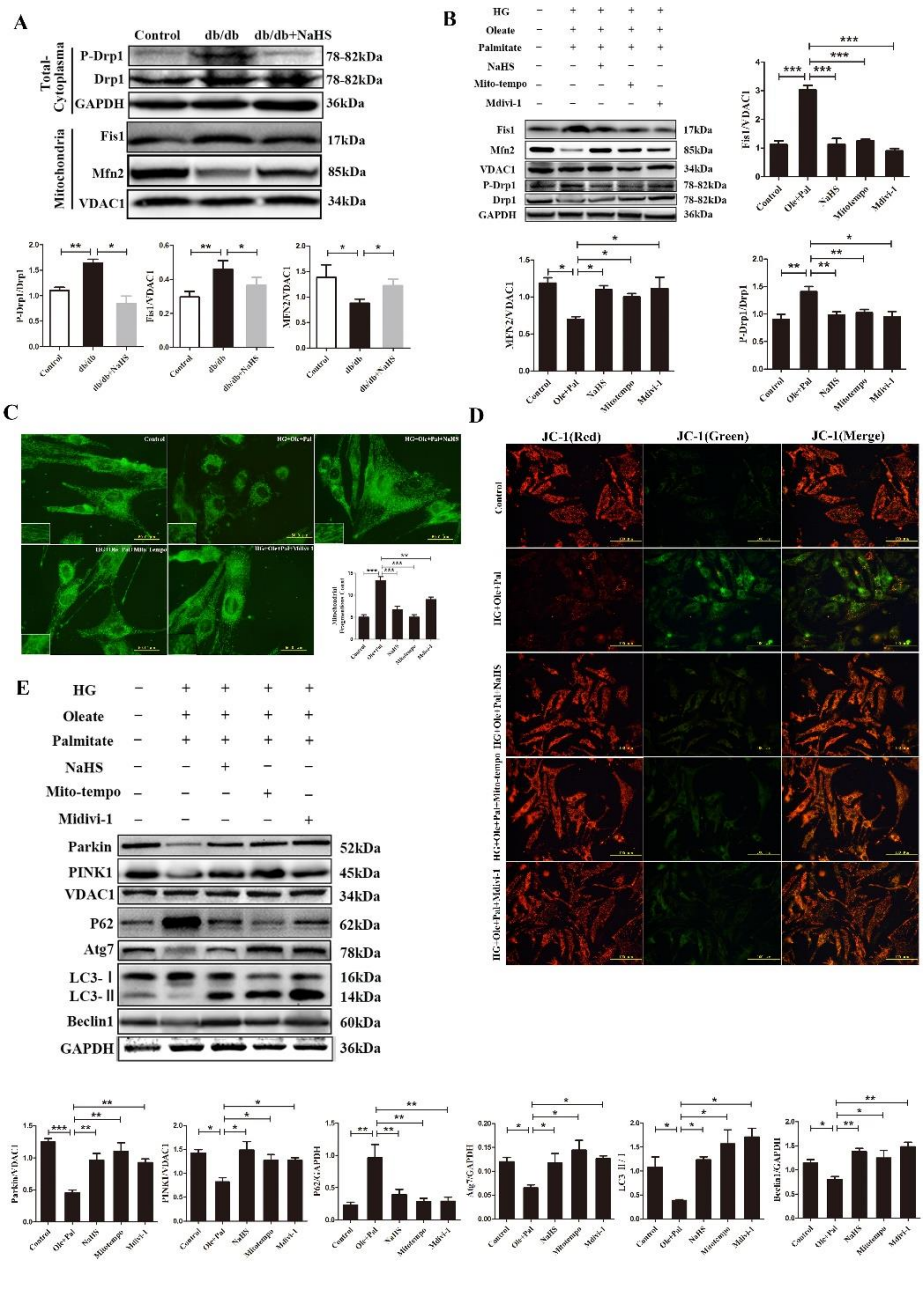
Exogenous H_2_S protected mitochondria by maintaining of mitochondrial dynamics. (A) The expression levels of the mitochondrial dynamics-related proteins, P-Drp1/Drp1, Fis1 and Mfn2, were measured in cardiac mitochondria by Western blotting. (B) The expression levels of the mitochondrial dynamics-related proteins, P-Drp1/Drp1, Fis1 and Mfn2, were examined in neonatal rat cardiomyocytes by Western blotting. (C) The mitochondrial morphology of neonatal rat cardiomyocytes was measured by MitoTracker green assay. (D) JC-1 assay was used to examine the mitochondrial membrane potential of neonatal rat cardiomyocytes. (E) The expression of Parkin, PINK1, Beclin1, Atg7, P62 and LC3II/I was detected in neonatal rat cardiomyocytes with Mito-Tempo and Midivi-1 treatment in by Western blotting. Values are presented as the mean ± S.D. from n = 5 replicates. *P<0.05, **P<0.01, ***P<0.001.

We assessed the effects of hyperglycemia and hyperlipidemia on H_2_S production in the cardiac tissues of db/db mice, our results showed that endogenous H_2_S levels in the hearts were decreased in db/db mice compared to both control and db/db mice treated with NaHS (Supplementary Fig. 2A). We also detected H_2_S levels in neonatal rat cardiomyocytes under hyperglycemia and hyperlipidemia by the H_2_S probe 7-azido-4-methylcoumarin (C-7Az). The H_2_S level was significantly decreased in the HG+Ole+Pal group, and its level was recovered following treatment with NaHS (Supplementary Fig. 2B).

We observed that the ejection fraction, left ventricular end-diastolic volume decreased and left ventricular mass were increased in db/db mice compared with control mice and db/db mice treated with NaHS. ExLVEDD (external left ventricular diastolic diameter) and ExLVESD (external left ventricular end-systolic dimension) were significantly increased in the db/db mice compared with control mice and decreased in the db/db mice treated with NaHS. Our data demonstrated that cardiac functions were not influenced in wild-type mice treated with NaHS (Supplementary Fig. 3).

**Figure 4.**
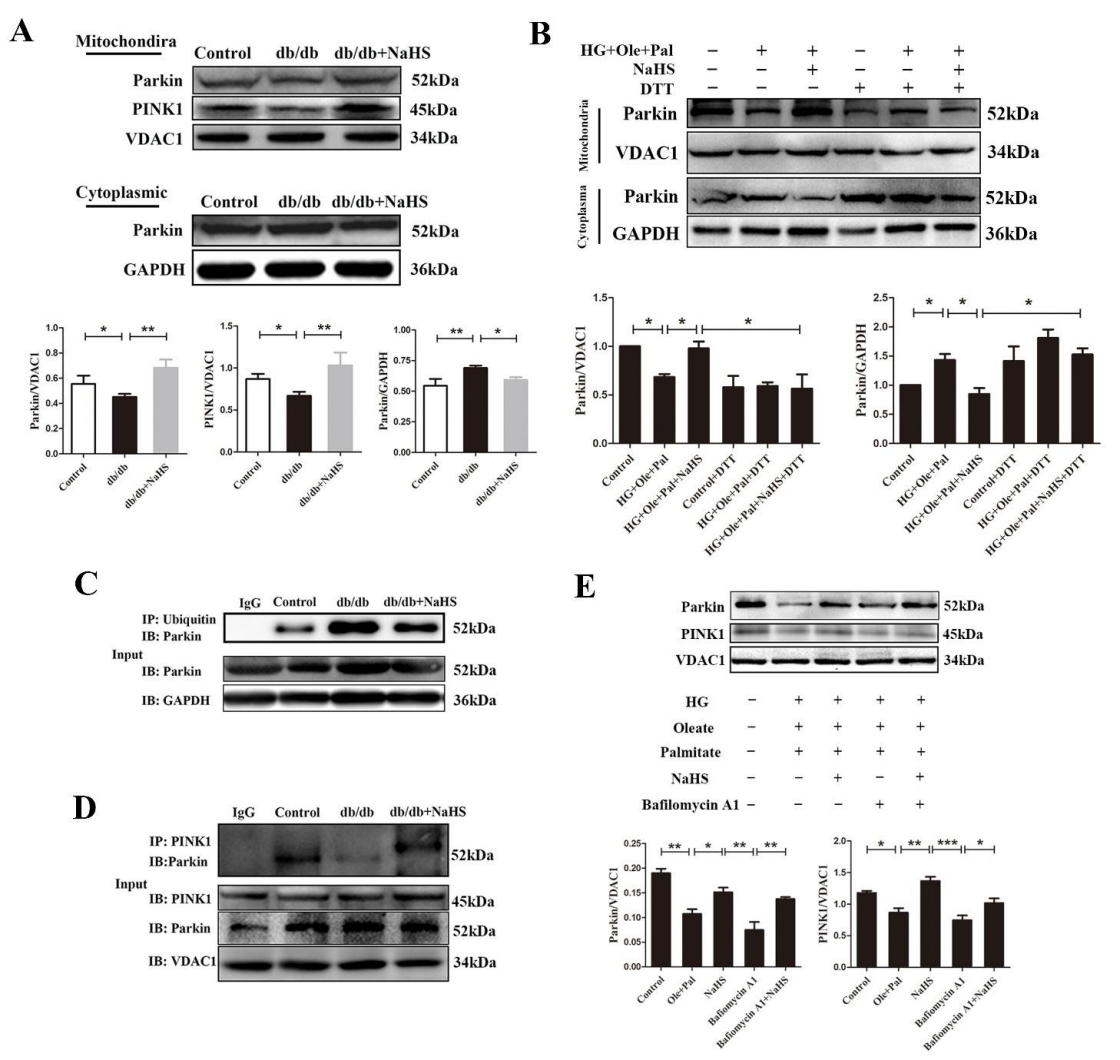
Exogenous H_2_S promoted mitophagy under hyperglycemia and hyperlipidemia. (A) Western blotting analysis and quantification of mitochondrial and cytoplasmic parkin protein in cardiac tissues. (B) Western blotting analysis and quantification of mitochondrial and cytoplasmic parkin protein in neonatal rat cardiomyocytes under hyperglycemia and hyperlipidemia. (C) The ubiquitination level of cytosolic parkin in cardiac tissues was examined by immunoprecipitation. (D) Immunoprecipitation assay was used to examine the interaction between PINK1 and parkin in cardiac tissues. (E) Western blot analysis detected the expression of parkin and PINK1 in the mitochondria of neonatal rat cardiomyocytes. Values are presented as the mean ± S.D. from n = 5 replicates. *P<0.05, **P<0.01, ***P<0.001.

### Exogenous H_2_S promoted mitophagy in cardiomyocytes under hyperglycemia and hyperlipidemia

To reveal whether exogenous H_2_S protects cardiac mitochondrial structure in type 2 diabetes, we examined mitochondrial morphology by TEM (Fig. 1). In the db/db mice group, disordered arrangement of myofilament, loss of cristae and mitochondrial swelling were observed. However, exogenous H_2_S ameliorated the abnormalities in mitochondrial morphology. The prominent morphological change in the cardiac tissues of db/db mice after NaHS injection was the formation of autophagic vacuoles that enveloped the cytoplasm, mitochondria and endoplasmic reticulum. Double membranes, giant autophagosomes filled with degraded organelles and autolysosomes were also observed (Fig. 1A, red arrows).

**Figure 5.**
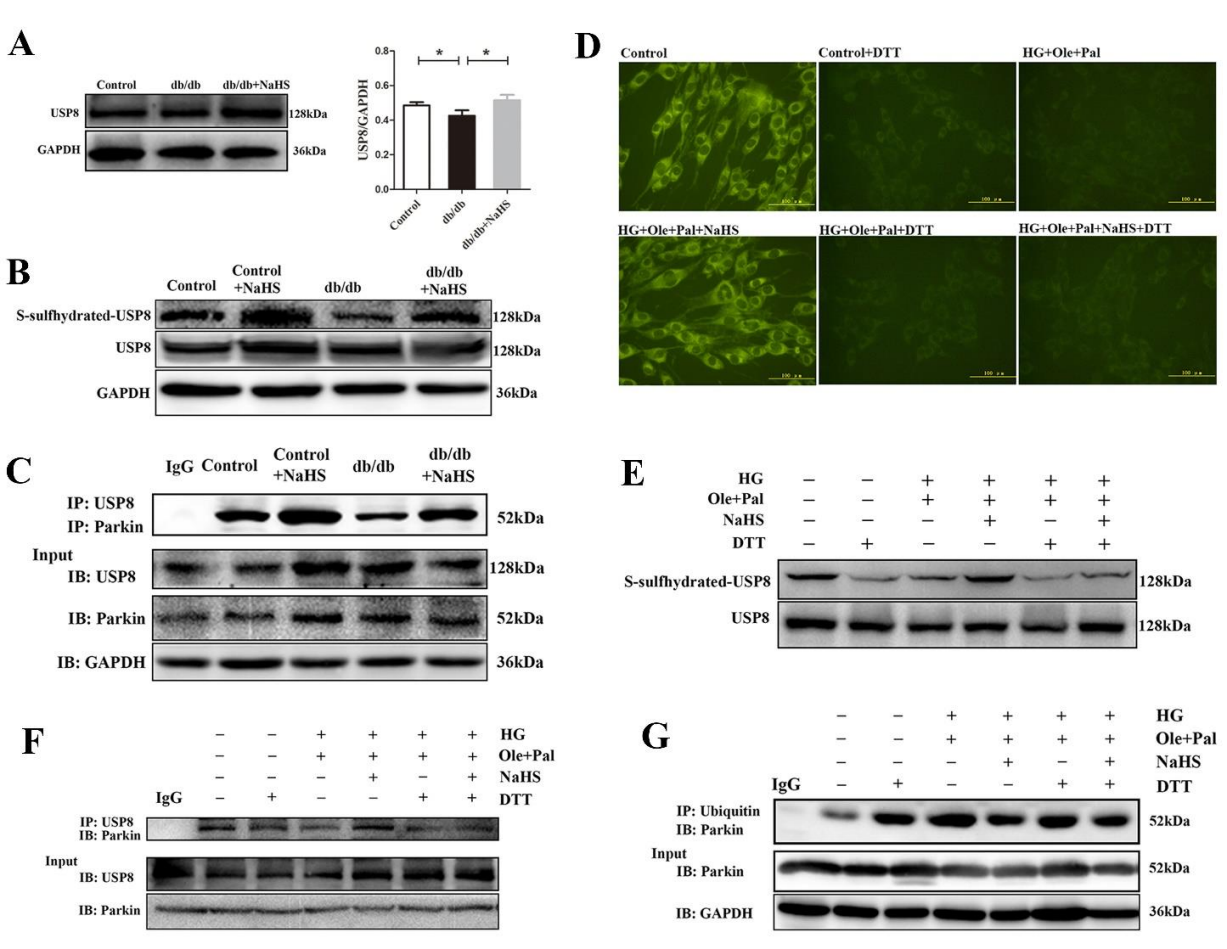
Exogenous H_2_S regulated the recruitment of parkin into mitochondria by the S-sulfhydration of USP8 in cardiomyocytes under hyperglycemia and hyperlipidemia. (A) The expression of USP8 in cardiac tissues. (B) The S-sulfhydration of USP8 in cardiac tissues was examined with the biotin switch (S-sulfhydration) method. (C) Immunoprecipitation assay was used to examine the interaction between USP8 and parkin in cardiac tissues. (D) Intracellular levels of polysulfide in neonatal rat cardiomyocytes were examined by a fluorescent probe, SSP4. (E) Neonatal rat cardiomyocytes were treated with dithiothreitol (DTT, 1mM, 10 min) or high glucose (40 mM), oleate (200 μM) and palmitate (200 μM) in the presence or absence of NaHS (100 μM) for 48 h. S-sulfhydration on USP8 were examined with the Biotin switch(S- sulfhydration) method. (F) Immunoprecipitation assay was used to examine interaction between USP8 and parkin in neonatal rat cardiomyocytes treated with DTT. (G) The ubiquitination level of cytosolic parkin in neonatal rat cardiomyocytes was measured by immunoprecipitation. Values are presented as the mean ± S.D. from n = 4 replicates. *P<0.05.

We also counted the number of autophagosomes and found that the number was decreased in the cardiac tissues of db/db mice compared to those of the control group and the db/db-NaHS group (Fig. 1B). These results suggested that exogenous H_2_S might exert cardioprotection by enhancing autophagy. To further reveal the effect of exogenous H_2_S on autophagy, we examined the expression of Atg7, Beclin1, P62 and LC3II/I in cardiac tissues (Fig. 1C) and neonatal rat cardiomyocytes (Fig. 1D). Our results showed that the expression of P62 was increased in db/db cardiac tissues and HG+Ole+Pal group compared with those of the control group, whereas exogenous H_2_S reduced the expression of P62 and increased the expressions of Atg7, Beclin1 and LC3II, which were related to the disruption of autophagy and lysosome bonding in cardiomyocytes under hyper-glycemia and hyperlipidemia. Bafilomycin A1 is a macrolide antibiotic that was characterized initially for its selective inhibition of vacuolar-type proton ATPase. This disruption prevents the fusion of autophagosomes with lysosomes, resulting in the accumulation of autophagosomes. Our results showed that the expression of LC3II was decreased following Bafilomycin A1 treatment, but treatment with NaHS increased the expression of LC3II under the Bafilomycin A1 treatment conditions. These results suggested that the exogenous H_2_S-induced increase of LC3Ⅱ levels may be involved in promoting autophagic vacuole formation rather than reducting of lysosome degradation.

Next, we evaluated autophagy by MDC staining, which can label acidic endosomes, lysosomes and autophagosomes, resulting in visible green patches of fluorescence. Our results showed that autophagy was increased in HG+Ole+Pal+NaHS group compared with HG+Ole+Pal group (Fig. 1E). Next, we further detected that compared to HG+Ole+Pal group and bafilomycin A1 groups, exogenous H_2_S-treated groups exhibited increased the formation of mitophagosomes (red fluorescence) and increased fusion of damaged mitochondria with lysosomes (green fluorescence) as evidenced by mitophagy dye and lysosome dye, respectively (Fig. 2A). Previous studies have demonstrated that LC3B translocated to mitochondria, resulting in the targeted removal of damaged mitochondria through the action of lysosomes [19]. Our results showed that the expression of LC3B was decreased in the cardiac mitochondria of db/db mice and neonatal rat cardiomyocytes treated with high glucose, oleate and palmitate (Fig. 2B and C). These results indicated that exogenous H_2_S promoted mitophagy in cardiomyocytes under hyperglycemia and hyperlipidemia.

### Exogenous H_2_S improved cardiac mitochondrial function and inhibited mitochondrial apoptotic pathways

To further investigate the effects of H_2_S on mitochondrial function, we also examined the activities of mitochondrial respiration chain complexes I, II and V which were impaired in db/db cardiac tissues compared with those of control mice, whereas NaHS ameliorated the activities of these complexes in the cardiac tissues of db/db mice (Supplementary Fig. 4A-C). We also measured mitochondrial respiratory functions, including the respiration of state 3 and 4, the respiratory control rate (RCR) and the ADP/O ratio to reflect mitochondrial respiration chain activities. Our results showed that the state 3, the RCR and the ADP/O ratio of mitochondria were significantly decreased in db/db mice compared with NaHS-treated and control mice (Supplementary Fig. 4D-G). Adenosine triphosphate (ATP) production through the respiratory chain is accompanied by the production of ROS as a result of electron leakage from the electron transport chain. It has been reported that oxidative stress can aggravate diabetic cardiomyopathy (DCM). Our results showed that the activities and expression of SOD and CAT, ROS scavenging enzymes and the ratio of GSH/GSSH were all higher in the cardiac tissues of the NaHS group than in those of the db/db group (Supplementary Fig. 4H). The treatment of neonatal rat cardiomyocytes with high glucose, palmitate and oleate mimicing type 2 diabetes cardiomyopathy in vitro, led to increase ROS production, and this effect was inhibited by exogenous H_2_S. The Mito-Sox, DCFH-DA and DHE were used to measure mitochondrial ROS, cellular superoxide anion and cytoplasmic H_2_O_2_, respectively (Supplementary Fig. 5A-C). The expression of Mn-SOD was downregulated in HG+Ole+Pal group compared with the control group, whereas it was upregulated by NaHS treatment (Supplementary Fig. 5D).

To demonstrate whether exogenous H_2_S inhibited the mitochondrial apoptotic pathway, we examined the expression of Cl-caspase 9, cytC, Bax and Bcl2 (Supplementary Fig. 6). Our results showed that the expression of Cl-caspase 9 and cytC in the cytoplasma and the expression of Bax in mitochondria were increased, while the expression of Bcl2 and cytC in mitochondria was decreased in db/db mice compared with control mice and NaHS -treated mice (Supplementary Fig. 6A). We also examined the anti-apoptotic effect of exogenous H_2_S on neonatal rat cardiomyocytes under hyperglycemia and hyperlipidemia state (Supplementary Fig. 6B), the results were consistent with the presence in vivo anti-oxdiation in db/db mice after treatment with exogenous H_2_S.

### Exogenous H_2_S regulated mitochondrial fission/fusion

Some studies revealed that increases in mitochondrial fusion are thought to be the protective mechanism against oxidative stress [5, 20]. Excessive mitochondria-derived ROS eventually accelerate aging, neurodegenerative disorders, and cardiovascular diseases. Mitochondria are highly dynamic organelles and their morphology continuously changes through fusion and fission [21]. To further investigate how exogenous H_2_S regulated mitochondrial morphology, the Drp1, Fis1 and mitofusin (Mfn2) involved in mitochondrial dynamics, were examined in cardiac tissues and neonatal rat cardiomyocytes by western blot. Our results showed that P-Drp1/Drp1 and Fis1 associated with mitochondrial fission, were increased in db/db cardiac tissues and under HG+Ole+Pal condition compared with controls, whereas these proteins were down-regulated in the NaHS groups (Figure 3A and B). The expression of Mfn2 decreased in the db/db mice and the HG+Ole+Pal group, compared with control group and NaHS group (Figure 3A and B). Mito-tracker assay was also used to examine the mitochondrial morphology of neonatal rat cardio-myocytes. The results showed more small mitochondria (diameter less than 2 μm) in HG+Ole+Pal group than in NaHS and Mito-tempo and Mdivi-1 groups (Fig. 3C). The impaired mitochondria indicate an alteration in mitochondrial membrane potential, which can be monitored by using JC-1. As shown in the results, the mitochondrial membrane potential was obviously altered in the HG+Ole+Pal group (the green fluorescence and red to green ratio were significantly decreased in the HG+Ole+Pal group compared with the control group) (Fig. 3D). The imbalance of fission and fusion leads to the generation of uneven mitochondria, the storage of oxidized and damaged proteins in mitochondria with lower membrane potential and the subsequently elimination of these mitochondria through mitophagy, the selective autophagic removal of mitochondria. To further investigate the effect of mitochondrial dynamics on mitophagy, we also examined the expression of Atg7, Beclin1, P62 and LC3 II/I under the treatment of Mdivi-1 and Mito-tempo. Our results suggested that Mdivi-1, Mito-tempo and exogenous H_2_S increased the expression of these proteins (Fig. 3E).

Taken together, these results suggested that exogenous H_2_S protected the intact morphology of intact mitochondria against hyperglycemia- and hyper-lipidemia- induced mitochondrial fission.

**Figure 6.**
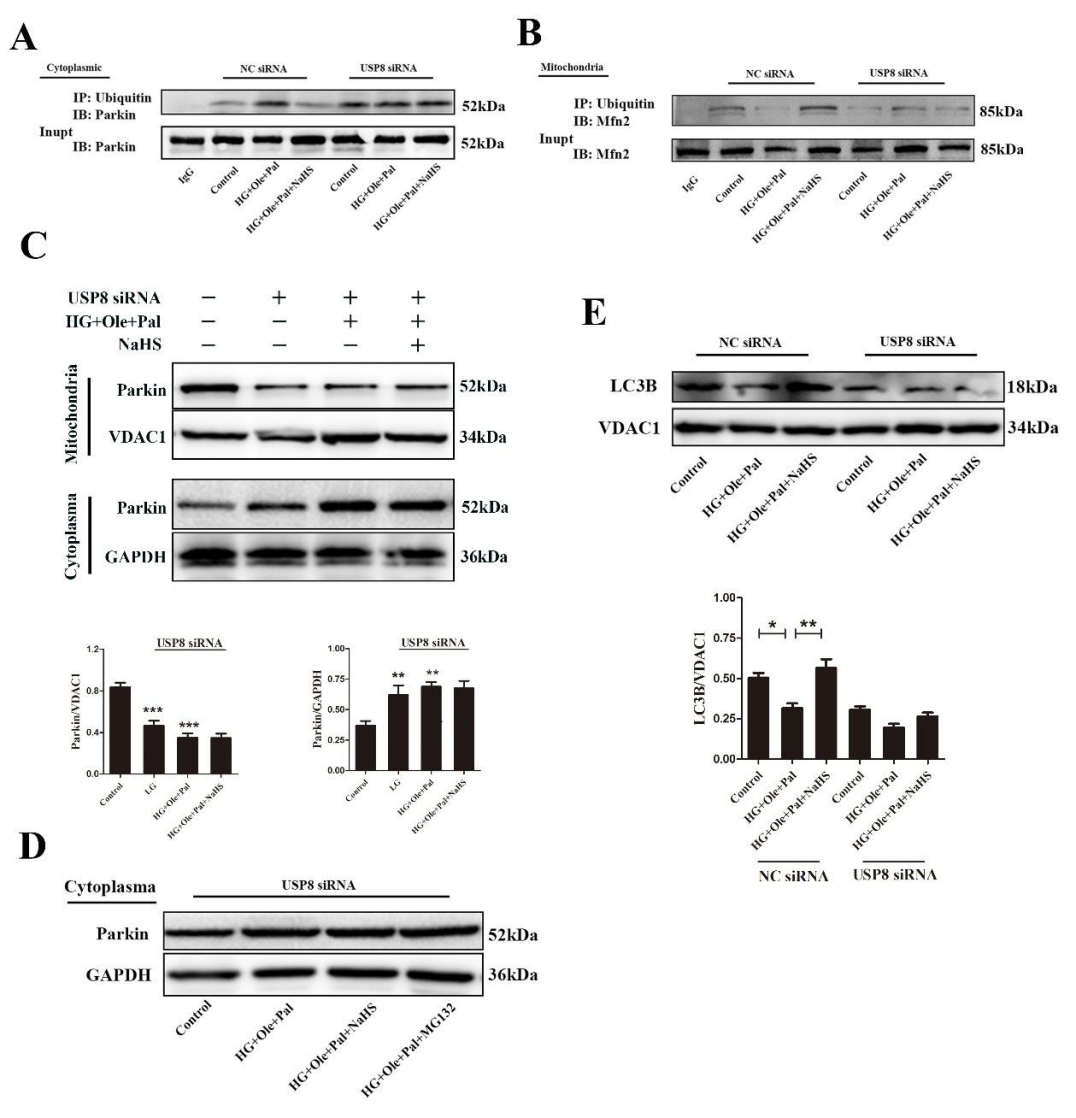
Exogenous H_2_S upregulated mitophagy through the activation of the USP8 signaling pathway. (A) The ubiquitination level of cytosolic parkin was examined following USP8 siRNA treatment by immunoprecipitation. (B) The ubiquitination level of mitochondrial Mfn2 was examined following USP8 siRNA treatment by immunoprecipitation. (C) Western blotting analysis and quantification of mitochondrial and cytoplasmic parkin protein under USP8 siRNA treatment. Values are presented as the mean ± S.D. from n = 4 replicates. **P<0.01vs Control, ***P<0.001 vs Control. (D) The expression of parkin in cytoplasma under USP8 siRNA and MG132 treatment. (E) Western blotting analysis of mitochondrial LC3B protein under USP8 siRNA treatment. Values are presented as the mean ± S.D. from n = 4 replicates. *P<0.05, **P<0.01.

### Exogenous H_2_S promoted mitophagy by the recruitment of parkin to mitochondria under hyperglycemia and hyperlipidemia

To investigate whether parkin is involved in the effects of H_2_S on mitophagy, we investigated its intracellular localization. Western blotting analysis of cytoplasmic and mitochondrial protein extracts indicated increased mitochondrial accumulation of parkin protein in cardiac tissues of db/db treated with H_2_S compared with those of db/db mice (Fig. 4A). We performed western blot analyses of cytoplasmic and mitochondrial protein extracts, and the results further indicated the mitochondrial accumulation of parkin protein. The purity of the mitochondria was quantified by western blotting analysis of proteins from different cellular organelles. The results also revealed that the mitochondrial contaminants were largely removed from the total-cytoplasm extracts (Supplementary Fig. 7A). Our results showed that the expression of parkin was decreased in mitochondria, but its expression of parkin in the cytoplasma remove of mitochondria was increased under hyperglycemia and hyperlipidemia (Fig. 4B). Parkin E3 ubiquitin-ligase activity is critical for the elimination of dysfunctional mitochondria by mitophagy [22]. We found that the cytosolic ubiquitination level of parkin was increased in db/db mice compared with the control mice and the NaHS-treated mice (Fig. 4C). The combination of PINK1 and parkin plays a pivotal role in mitophagy, and the interaction between PINK1 and parkin can be measured by immunoprecipitation assay [23]. We found that exogenous H_2_S enhanced the interaction between PINK1 and parkin compared with db/db cardiac tissues (Fig. 3D). We also measured the expression levels of parkin and PINK1 in mitochondria of neonatal rat cardiomyocytes and found that the expression of these proteins was decreased in HG+Ole+Pal group compared with control group and the NaHS group (Fig. 4E). All these results revealed that exogenous H_2_S promoted mitophagy by recruiting parkin in mitochondria under hyperglycemia and hyperlipidemia.

### Exogenous H_2_S regulated the recruitment of parkin into mitochondria by the S-sulfhydration of USP8 in cardiomyocytes under hyperglycemia and hyperlipidemia

Some evidences confirmed that the activation of parkin via ubiquitination can be regulated by DUB that may stabilize the basal level of active parkin. It has been proposed that USP8, a DUB associated with the removal of Ub conjugates from parkin. Our data showed that the expression of USP8 was decreased in cardiac tissues of db/db mice compared with those of control mice (Fig. 5A). S-sulfhydration, the addition of one sulfhydryl to the thiol side of the cysteine residue and the formation of a persulfide group (R-S-S-H), has been reported as a novel posttranslational modification by H_2_S in eukaryotic cells [24]. Our data showed that the sulfhydration level of USP8 was increased in NaHS-treated db/db mice compared with db/db mice (Fig. 5B). Exogenous H_2_S had no significant effects on the sulfhydration level of USP8 in the NaHS-treated control group with the untreated control group. To further explore the mechanisms of parkin activation, we immunoprecipitated cardiac lysate samples using an anti-USP8 antibody and blotted for parkin. The results showed that NaHS treatment increased the USP8/parkin interaction in db/db cardiac tissues (Fig. 5C).

Given the marked in vivo effects of exogenous H_2_S on the regulation of the sulfhydration level of USP8 in cardiac tissues, we conducted in vivo studies to investigate the role of exogenous H_2_S action in neonatal rat cardiomyocytes. We examined the intracellular production of polysulfide using a newly developed fluorescent probe, SSP4, a polysulfide sensitive fluorescent probe. Our results showed that NaHS could increase the SSP4 fluorescence intensity, suggesting the promotion of the polysulfide production (Fig. 5D). DTT is a kind of the reducing agent that can reverse the covalent modification in sulfhydration [25]. Our results showed that the effects of exogenous H_2_S on USP8 deubiquitylation and the interaction of USP8 with parkin were blocked with DTT treatment (Fig. 5E and F). We also examined the cytosolic ubiquitination level of parkin under DTT treatment. We found that DTT nearly inhibited the effect of exogenous H_2_S on the S-sulfhydration of USP8 and removing of Ub from parkin (Fig. 5E and G). In addition, we also detected the mitophagy formation under DTT treatment. Our results showed that DTT impaired the formation of mitophagy under control condition and HG+Ole+Pal+NaHS condition (Supplementary Fig. 7C). We speculated that exogenous H_2_S promoted the USP8-mediated deubiquitination of parkin by S-sulfhydration.

### Exogenous H_2_S upregulated mitophagy by activating the USP8/parkin signaling pathway

Given that silencing USP8 impeded parkin recruitment to mitochondria, we investigated whether it also affected mitophagy. H9c2 cells were transfected with either nontargeted or USP8 siRNA and treated with high glucose, oleate and palmitate for 48 h (Supplementary Fig. 7B). We noticed an apparent increase in the levels of ubiquitination level on parkin in the absence of USP8 (Figure 6A). Mfn2 is a key protein regulating mitophagy. The ubiquitination of Mfn2 by parkin plays an important role in regulating mitophagy. We detected the ubiquitination level of Mfn2 by immunopreipitation, which showed that exogenous H_2_S could increase the ubiquitination level of Mfn2 under hyperglycemia and hyperlipidemia (Fig. 6B). Whereas, with treatment of exogenous H_2_S did not increase the ubiquitination level of Mfn2 under treatment of USP8 siRNA. Next, we examined the expression level of parkin under treatment of USP8 siRNA in mitochondria and cytoplama without mitochondria. Our results showed that the expression of parkin was decreased in mitochondria but increased in cytoplasma under treatment of USP8 siRNA (Fig. 6C). In addition, we also added MG-132 under the USP8 siRNA condition. MG132 is a kind of proteasome inhibitor. Our results showed that the expression of parkin was not significantly changed in USP8 siRNA +MG-132 group compared with USP8 siRNA group (Fig. 6D). The increased translocation of parkin into mitochondria did not mostly result from increased expression of parkin. Compared with control cells, the cells transfected with USP8 siRNA showed that decreased expression of LC3B in mitochondria under different treatment conditions (Fig. 6E). Taken together, these findings further support a model whereby the USP8-mediated deubiquitination of parkin is critical for mitophagy, which ameliorates mitochondrial morphology in diabetic cardiomyopathy.

**Figure 7.**
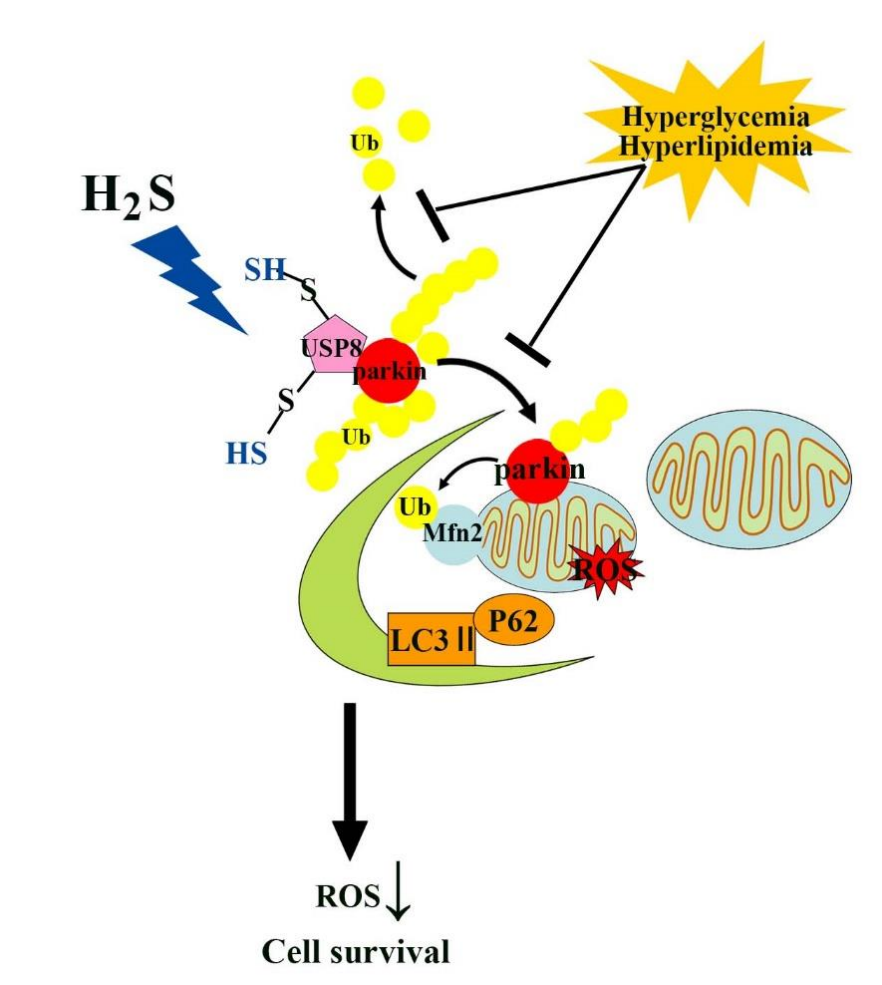
The role of H_2_S in the regulation of cardiac mitophagy by the S-sulfhydration of USP8 in a type 2 diabetes model.

## DISCUSSION

Recent studies have suggested that impaired mitochondria may contribute to an increased risk of diabetic cardiomyopathy [26]. Our results indicated that (i) exogenous H_2_S could attenuate mitochondrial fragments induced by hyperglycemia and hyperlipidemia in cardiomyocytes; (ii) exogenous H_2_S promoted the recruitment of parkin in mitochondria; and (iii) exogenous H_2_S promoted USP8 expression and the S-sulfhydration of USP8 facilitated the deubiquitination of parkin, enhancing mitophagy in cardiomyocytes.

Emerging experimental and clinical evidence indicates that alterations in H_2_S bioavailability play a prominent role in diabetes [27]. Our study showed that the administration of NaHS markedly improved cardiac function in db/db mice. There was no significant difference in cardiac function between the group treated with NaHS alone and the control group indicating that the administration of NaHS alone did not cause any damage to cardiac function. Taken together, these findings suggest that altered of H_2_S content might be associated with diabetic cardiomyopathy.

Cardiac function is highly dependent on the oxidative energy generated by mitochondria, and mitochondria are susceptible to oxidative damage [28]. Previous studies found that a characteristic of db/db mice was the accumulation of intramyocardial lipid metabolites, which caused additional ROS production and then damaged the function of mitochondria. Our study showed that the increase in cardiac ROS was accompanied by impaired mitochondrial function and altered mitochondrial morphology in db/db mice, and this funding was further confirmed in HG+Ole+Pal-treated cardiomyocytes. H_2_S has been shown to have powerful antioxidant properties.

It has been fully proved that ROS is tightly related to the aging process and mitochondrial dysfunction plays a pivotal role in ROS production [29-31]. According to some studies, decreased H_2_S levels are closely related to aging. Plasma H_2_S levels have been reported to decline in an age-dependent manner in human subjects aged 50-80 years, and some studies demonstrated that exogenous H_2_S contributed to the defense against oxidative damage and mitochondria protection [32]. A recent study reported that exogenous H_2_S attenuated the aging process via the PI3K/AKT and CaMKKβ/AMPK pathways [33]. In aging cells, damaged mitochondria and proteins accumulate which are toxic to cells and increase ROS production[34]. Mitophagy is an effective way to eliminate dysfunctional mitochondria. Our present study speculated that H_2_S decreased ROS production by regulating mitochondrial function in the hearts of db/db mice, which might be a way to resist senescence.

Mitochondrial dysfunction is becoming an increasingly common cause of type2 diabetes and insulin resistance [35]. Increases in mitochondrial fusion followed by fission events are thought to be a protective mechanism against oxidative stress during development, during the cell cycle, or in response to various cytotoxic conditions [36, 37]. However, oxidative stress can result in excessive mitochondrial fission, contributing to mitochondrial dysfunction[38]. Our results showed that mitochondrial fragmentation increased under hyperglycemia and hyperlipidemia and treatment with NaHS could decrease mitochondrial fission. Drp1 is predominantly distributed in the cytoplasm and associates with the mitochondrial outer membrane. The Fis1 protein is localized to the outer mitochondrial membrane via a C-terminal transmembrane domain. It has been reported that the overexpression of Fis1 induces mitochondrial fragmentation and that a portion of cytosolic Drp1 can be recruited to mitochondria through an interaction with Fis1 [21]. Mfn2 localizes to the outer membrane of mitochondria and mediates mitochondrial fusion. In our study, we demonstrated that the expression of Fis1 and P-Drp1 was increased and that the expression of Mfn2 was decreased in the cardiac tissues of db/db mice compared with those of control mice, whereas exogenous H_2_S could decrease mitochondrial fission. In accordance with these findings, we observed that H_2_S decreased mitochondrial fission in cardiomyocytes under hyperglycemia and hyper-lipidemia.

Mitochondrial integrity is critically regulated by autophagic clearance, a specialized type of autophagy [39]. Autophagic pathways are an important protection system against the ROS-mediated damage of proteins and organelles in the cell [40]. Moreover, increasing evidence has demonstrated that basal levels of autophagy are required for normal heart function[41]. Our study observed that the level of autophagy was decreased in cardiac tissues of db/db mice compared with those of control mice, and that H_2_S could promote cardiac autophagy. Furthermore, our results indicated that exogenous H_2_S could facilitate the clearance of impaired mitochondria. Hence, our study focused on how H_2_S regulates mitophagy in the hearts of db/db mice.

Parkin is an RBR-type E3 ligase that normally localizes in the cytosol as an autoinhibited form [42]. In 2008, Richard Youle *et al*. reported that cytosolic parkin was recruited to damaged mitochondria to be degradaed through an autophagy pathway, which undoubtedly opened a new research field termed parkin-mediated mitophagy [43]. Once parkin reaches the mitochondrial outer membrane, its E3 activity is fully activated and various mitochondrial outer membrane proteins are ubiquitinated. In our study, we found that exogenous H_2_S activated parkin and then promoted the ubiquitination of Mfn2, which recruits parkin to mitochondria [44, 45]. The regulation of parkin by autoubiquitination has the potential to profoundly affect its function [7]. Autoubiquitination can be antagonized by DUBs, which remove Ub from the E3. Several DUBs that counteract parkin E3 Ub ligases by catalyzing the removal of Ub from substrates have been reported to regulate mitophagy [46, 47]. USP8, a DUB not previously implicated in mitochondrial quality control, is critical for parkin-mediated mitophagy. Our results showed that hyperglycemia and hyperlipidemia decreased the translocation of parkin in the mitochondrial outer membrane and decreased the interaction between USP8 and parkin. Treatment with exogenous H_2_S increased the mitochondrial translocation of parkin. Activated parkin then promotes the ubiquitination of Mfn2, which recruits parkin to mitochondria [44]. We found that exogenous H_2_S increased the ubiquitination level of Mfn2, which also indicated that exogenous H_2_S promoted mitophagy. These alterations promoted the survival of cardiocytes in response to oxidative stress. However, the mechanism of H_2_S is unknown. H_2_S has been recently demonstrated to posttranslational modification of proteins by the formation of a persulfide (-SSH) bond with the reactive cysteine residues of target proteins, termed as S-sulfhydration. After *S*-sulfhydration, proteins change their original function, serving as important switchers or regulators [48]. It is important to note that H_2_S induces S-sulfhydration on cysteine thiols under oxidation conditions. We speculated that exogenous H_2_S likely ameliorated mitochondrial morphology by influencing the function of USP8 by S-sulfhydration and thereby regulating mitophagy. Our results showed that exogenous H_2_S could result in the S-sulfhydration of USP8 under hyperglycemia and hyperlipidemia, leading to the enhanced interaction of USP8 with parkin and the translocation of parkin into mitochondria. Therefore, DTT was used to inhibit the sulfhydration modification. We found that DTT nearly abrogated the effect of exogenous H_2_S on the translocation of parkin in mitochondria. Further, DTT inhibited the interaction of USP8 with parkin. To further investigate the effect of the regulation of USP8 by exogenous H_2_S on mitophagy, we knocked down of USP8 with siRNA and found that this treatment impaired the recruitment of parkin to damaged mitochondria and increased the ubiquitination level of parkin. Regardless of the precise mechanism, our work uncovers a novel layer of the exogenous H_2_S-mediated regulation of diabetic cardiomyopathy through the S-sulfhydration of USP8, critical for parkin-dependent mitophagy (Fig. 7).

### Conclusions

In summary, our results provide definitive evidence that H_2_S could ameliorate cardiac impairment in db/db mice and improve hyperglycemia- and hyperlipidemia-induced injury in neonatal rat cardiomyocytes. This protective effect of H_2_S could partly be attributed to activation of USP8 via S-sulfhydration, which might contribute to the improved translocation of parkin in mitochondria and promote mitophagy. The above evidence provides new insight into the mechanisms responsible for the antioxidative effects of H_2_S in the context of diabetic cardiomyopathy.

### Limitation

In this study, we showed that H_2_S upregulated USP8 and increased the translocation of parkin into mitochondria. However, it is unclear how H_2_S controls the expression of USP8. Some studies demonstrated that specific protein 1 (Sp1) decreased USP8 transcription. Huang et al. revealed that inhibited sp1 expression by activating miR-145 [49]. In our future study, we need to address the molecular mechanism which H_2_S modulates USP8.

## Supplementary Materials

The Supplemenantry data can be found online at: www.aginganddisease.org/EN/10.14336/AD.2019.0524.
