# Supplementary material for: Exogenous H2S Promoted USP8 Sulfhydration to Regulate Mitophagy in the Hearts of db/db Mice
Source: Aging Dis. 2020 Mar 9;11(2):269–85. doi: 10.14336/AD.2019.0524 (PMC7069468; doi:10.14336/AD.2019.0524)
Supplement: Supplementary file 1 — The Supplemenantry data can be found online at: www.aginganddisease.org/EN/10.14336/AD.2019.0524. [file AD-11-2-269-s.pdf]

SUPPLEMENTARY DATA

Exogenous H_2S Promoted USP8 Sulfhydration to Regulate Mitophagy in the Hearts of db/db Mice

Yu Sun[1,#], Fanghao Lu[1,#], Xiangjing Yu[1], Bingzhu Wang[1], Jian Chen[1], Fangping Lu[1], Shuo Peng[1], Xiaojiao Sun[1], Miao Yu[1], He Chen[2], Yan Wang[3], Linxue Zhang[1], Ning Liu[1], Haining Du[1], Dechao Zhao[4,*] , Weihua Zhang[1,5,*]

[1]Department of Pathophysiology, Harbin Medical University, Harbin, China. [2]Department of Forensic Medicine, Harbin Medical University, Harbin, China. [3]Department of Urologic Surgery, First affiliated hospital of Harbin Medical University, Harbin, China. [4]Department of Cardiology, First affiliated hospital of Harbin Medical University, Harbin, China. [5]Key Laboratory of Cardiovascular Medicine Research (Harbin Medical University), Ministry of Education, Harbin, China

© 2019. Sun Y et al. Published online at http://www.aginganddisease.org/EN/10.14336/AD.2019.0524

SUPPLEMENTARY DATA

SUPPLEMENTARY METHODS

Mitochondrial ROS and cellular ROS level analysis

Mitochondrial ROS production was measured using the Mito-SOX Red mitochondrial superoxide indicator (Invitrogen). The neonatal rat cardiomyocytes were loaded with 5 μM Mito-SOX Red at 37℃ for 15 min. Red fluorescence was measured at 583 nm following excitation at 488 nm using a fluorescence microscope (Olympus, XSZ-D2). The oxidative radical levels were examined using the DCFH-DA (Beyotime, China) staining method based on the conversion of nonfluorescent DCFH-DA to the highly fluorescent DCF upon intracellular oxidation by ROS. Neonatal rat cardiomyocytes were seeded on coverslips and incubated (45 min, 37 °C, in the dark) in serum-free media containing DCFH-DA (10 μM). After incubation, the conversion of DCFH-DA to the fluorescent product DCF was measured using a fluorescence microscope (Olympus, XSZ-D2) with an excitation wavelength of 484 nm and an emission wavelength of 530 nm. Background fluorescence (conversion of DCFH-DA in the absence of cells) was corrected by the inclusion of parallel blanks. The level of intracellular superoxide anions was detected by dihydroethidium (DHE) (Beyotime, China). Neonatal rat cardiomyocytes were incubated in serum-free media-containing DHE at 37 °C for 30 min. DHE can be converted into ethidium by superoxide anions and it showed red fluorescence at 535 nm. The cells were then incubated with Hoechst 33342 to visualize nuclear localization.

Measurement SOD and CAT activity and glutathione content

Superoxide dismutase (SOD), catalase (CAT) and glutathione (GSH) in the supernatant were measured using a spectrophotometer (Jiancheng Institute of Bioengineering, Nanjing, China). All assays were conducted according to the kit instructions.

Measurement of serum and cardiac H_2S levels

The measurement of H_2S production in serum and isolated cardiac tissues followed the established protocol[1, 2]. Briefly, serum was mixed with 10% trichloroacetic acid. The reaction was stopped by 1% zinc acetate, followed by incubation with N,N-dimethyl-p-phenylenediamine sulfate (DPD) for 15 min. The absorbance at 670 nm was measured with a spectrophotometer.

Detection of H_2S by 7-azido-4-methylcoumarin

The fluorescence response of H_2S in cardiomyocytes was tested using 7-Azido-4-Methylcoumarin, (C-7Az, Sigma), which has been proven to selectively respond to H_2S[3]. Cardiomyocytes were incubated with 50 μmol/L C-7Az PBS for 30 min and then the cells were washed with PBS. The fluorescen response of C-7Az to H_2S in cardiomyocytes was visualized using fluorescence microscopy at and excitation wavelength of a 720 nm laser using a Zeiss LSM 510 inverted confocal microscope.

Glucose tolerance tests

Mice were intraperitoneally injected with D-glucose (2 g/kg mass). Tail blood was collected, and blood glucose was determined using a glucometer.

Echocardiographic analysis of cardiac function

The cardiac functions of mice were assessed using an echocardiography system (GE VIVID7 10S, USA) after twelve weeks of treatment. Echocardiography was performed on self-breathing mice under anesthesia (intraperitoneal injection of 1% pentobarbital sodium at 6 $mL\cdot kg^{-1}$ body weight).

Mitochondrial enzyme complexes

Complex I, II and V values were measured by using a spectrophotometer (GENMED, Shanghai, China). All assays were conducted according to the kit instructions.

[1] Sun A, Wang Y, Liu J, Yu X, Sun Y, Yang F, *et al.* (2016). Exogenous H2S modulates mitochondrial fusion-fission to inhibit vascular smooth muscle cell proliferation in a hyperglycemic state. Cell Biosci, 6:36.

© 2019. Sun Y et al. Published online at http://www.aginganddisease.org/EN/10.14336/AD.2019.0524

SUPPLEMENTARY DATA

[2] Wu L, Yang W, Jia X, Yang G, Duridanova D, Cao K, *et al.* (2009). Pancreatic islet overproduction of H2S and suppressed insulin release in Zucker diabetic rats. Lab Invest, 89:59-67.

[3] Chen B, Li W, Lv C, Zhao M, Jin H, Du J, *et al.* (2013). Fluorescent probe for highly selective and sensitive detection of hydrogen sulfide in living cells and cardiac tissues. Analyst, 138:946-951.

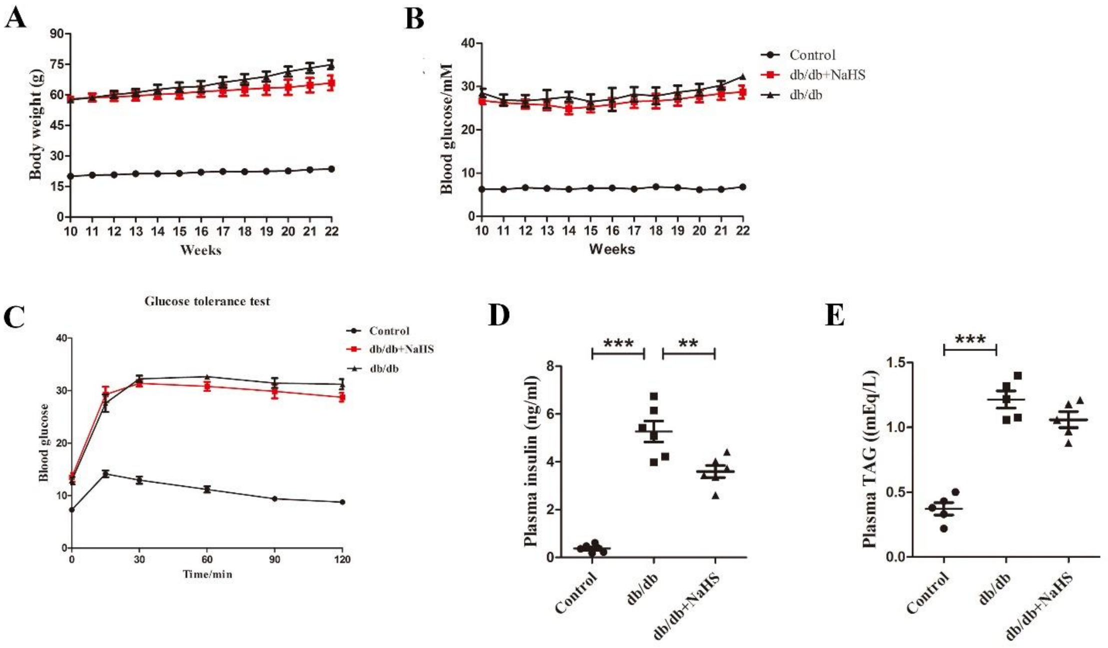

Supplementary Figure 1. General parameters were detected. **(A)** Body weight and **(B)** blood glucose were measured in 10- to 22-week-old control mice, db/db mice and db/db-NaHS mice (n=6). **(C)** Intraperitoneal glucose tolerance test results of 22-week-old control mice, db/db mice and db/db-NaHS mice. Mice were injected with 2 g glucose/kg body weight (n=6). **(D)** Plasma insulin content and **(E)** plasma triglycerides were measured in 22-week-old control mice, db/db mice and db/db-NaHS mice. Values are presented as the mean ± S.D. n=6, ***p*<0.01, ****p*<0.001.

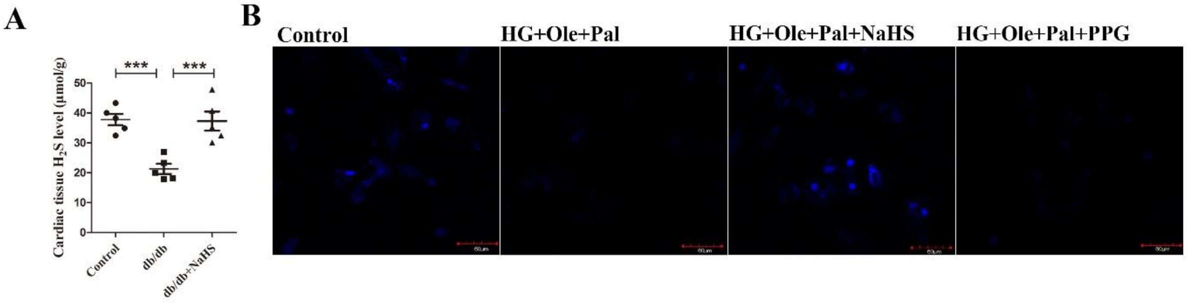

Supplementary Figure 2. (A) Cardiac H_2S levels in control mice, db/db mice and db/db-NaHS mice. n=5, ****p*<0.001. (B) The fluorescence of H_2S was observed by fluorescence microscopy.

© 2019. Sun Y et al. Published online at http://www.aginganddisease.org/EN/10.14336/AD.2019.0524

SUPPLEMENTARY DATA

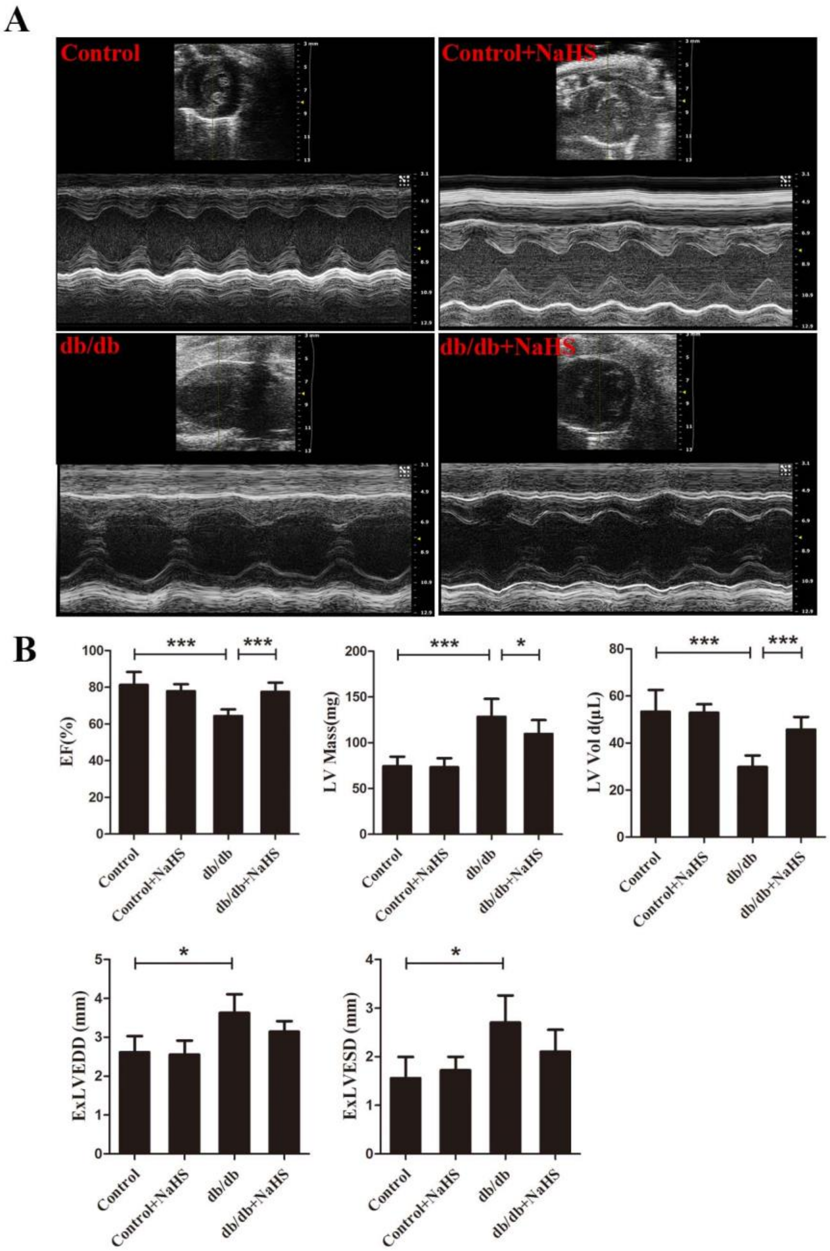

Supplementary Figure 3. Exogenous H_2S ameliorated heart function in db/db mice. (A) Respresentative M-mode echocardiographic images of the left ventricular diameter (LVD) in the control group, the control group treated with NaHS, the db/db group and the db/db group treated with NaHS. (**B**) Echocardiography showed ejection fraction (EF), left ventricular (LV) mass, left ventricular end-diastolic volume, external left ventricular diastolic diameter (ExLVEDD) and external left ventricular end-systolic dimension (ExLVESD). Values are presented as the mean ± S.D. from n = 4 replicates. **P*<0.05, ***P*<0.01, ****P*<0.001.

© 2019. Sun Y et al. Published online at http://www.aginganddisease.org/EN/10.14336/AD.2019.0524

SUPPLEMENTARY DATA

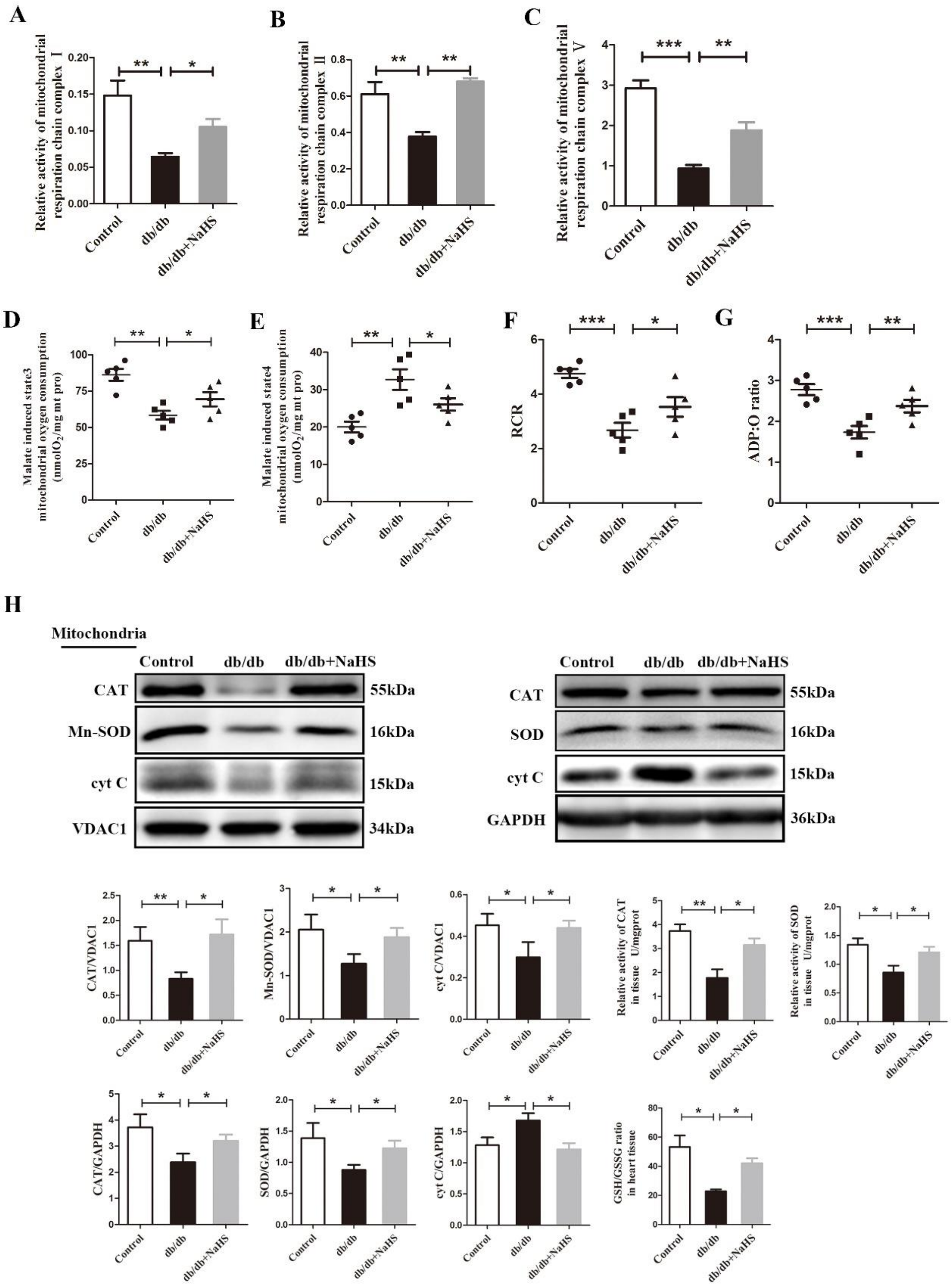

Supplementary Figure 4. Exogenous H_2S protected mitochondrial function and attenuated ROS production in db/db mice heart. (A-C) The activities of mitochondrial respiratory chain complexes I, II and V were examined in cardiac tissues using activity assay kits. (D and E) Mitochondrial state3 and 4 oxygen consumption, (F) the respiratory control rate (RCR) and (G) the ADP/O ratio were measured in db/db cardiac tissues. (H)The expression of CAT and SOD in the mitochondria and cytoplasm were detected by western blotting in cardiac tissues. The activities of CAT and SOD and the ratio of GSH/GSSH in cardiac tissues were detected using activity assay kits. Values are presented as the mean ± S.D. from n = 5 replicates. *P<0.05, **P<0.01, ***P<0.001.

© 2019. Sun Y et al. Published online at http://www.aginganddisease.org/EN/10.14336/AD.2019.0524

SUPPLEMENTARY DATA

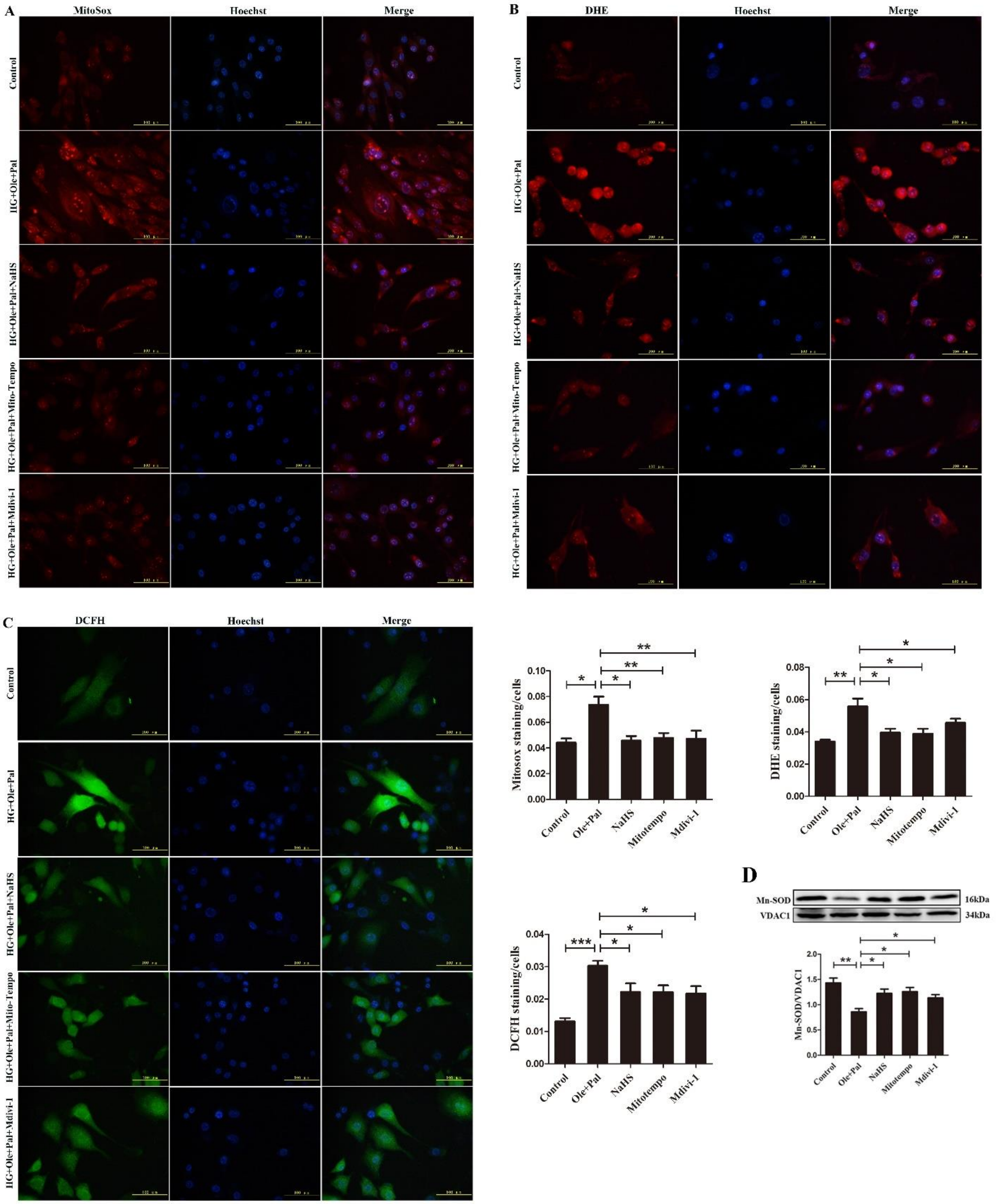

Supplementary Figure 5. **Exogenous H_2S suppressed the production of ROS in neonatal rat cardiomyocytes treated with high glucose, oleate and palmitate**. (A) Mitochondrial ROS levels were detected by Mitosox and nuclei were labeled by Hoechst (blue fluoresence). (B and C) Intracellular ROS levels were detected by DHE, DCFH-DA, and nuclei were labeled by Hoechst (blue fluoresence). (D) The expression of Mn-SOD in neonatal rat cardiomyocytes was measured. Values are presented as the mean ± S.D. from n = 5 replicates. **P*<0.05,***P*<0.01, ****P*<0.001.

© 2019. Sun Y et al. Published online at http://www.aginganddisease.org/EN/10.14336/AD.2019.0524

SUPPLEMENTARY DATA

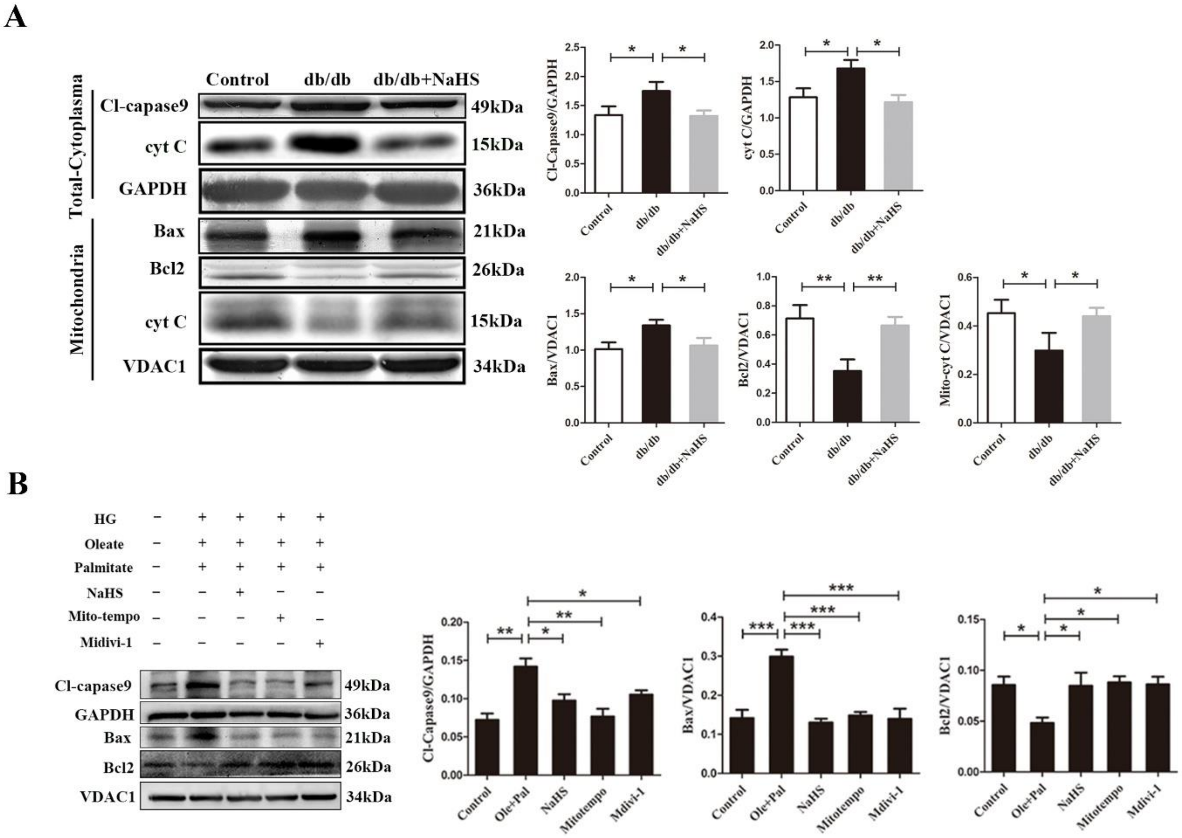

Supplementary Figure 6. **Exogenous H_2S inhibited mitochondrial apoptotic pathways.** (A) The expression of Cl-caspase9, Bax and Bcl2 was quantified in cardiac tissues by Western blotting. (B) The expression of Cl-caspase9, Bax and Bcl2 was detected in neonatal rat cardiomyocytes. Values are presented as the mean ± S.D. from n =5 replicates. **P*<0.05, ***P*<0.01, ****P*<0.001.

© 2019. Sun Y et al. Published online at http://www.aginganddisease.org/EN/10.14336/AD.2019.0524

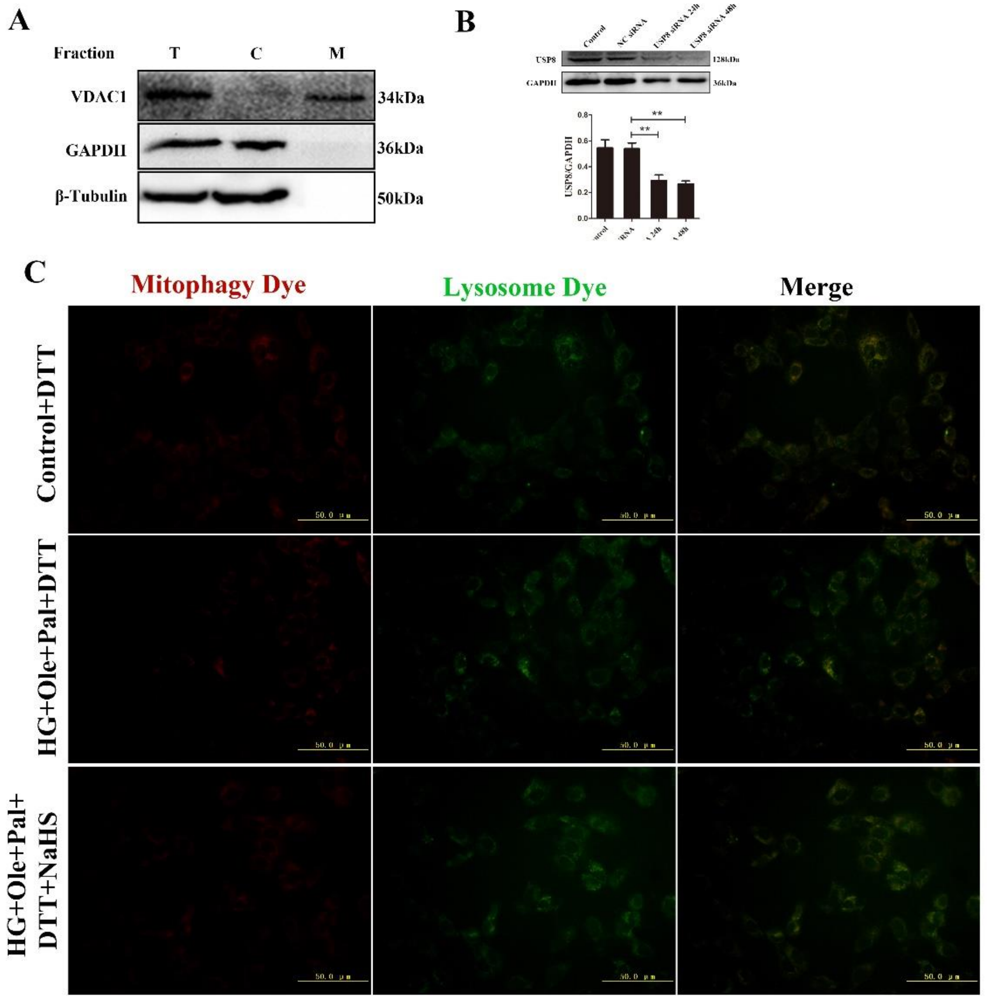

Supplementary Figure 7. (A) Immunoblotting analysis of protein markers was performed on isolated cellular organelles. (T: total-cytoplasma, C: cytoplasma without of mitochondria, M: mitochondria). (B)The expression of UPS8 after USP8 siRNA treatment. (C) Mitophagosomes were detected in neonatal rat cardiomyocytes treated with DTT in control, HG+Ole+Pal group. Values are presented as the mean ± S.D. from n = 4 replicates, ****P*<0.01

© 2019. Sun Y et al. Published online at http://www.aginganddisease.org/EN/10.14336/AD.2019.0524
